# CVnCoV and CV2CoV protect human ACE2 transgenic mice from ancestral B BavPat1 and emerging B.1.351 SARS-CoV-2

**DOI:** 10.1038/s41467-021-24339-7

**Published:** 2021-06-30

**Authors:** Donata Hoffmann, Björn Corleis, Susanne Rauch, Nicole Roth, Janine Mühe, Nico Joel Halwe, Lorenz Ulrich, Charlie Fricke, Jacob Schön, Anna Kraft, Angele Breithaupt, Kerstin Wernike, Anna Michelitsch, Franziska Sick, Claudia Wylezich, Bernd Hoffmann, Moritz Thran, Andreas Thess, Stefan O. Mueller, Thomas C. Mettenleiter, Benjamin Petsch, Anca Dorhoi, Martin Beer

**Affiliations:** 1 Institute of Diagnostic Virology, Friedrich-Loeffler-Institut, Greifswald-Insel Riems, Germany; 2 Institute of Immunology, Friedrich-Loeffler-Institut, Greifswald-Insel Riems, Germany; 3 CureVac AG, Tübingen, Germany; 4 Department of Experimental Animal Facilities and Biorisk Management, Friedrich-Loeffler-Institut, Greifswald-Insel Riems, Germany; 5 Friedrich-Loeffler-Institut, Federal Research Institute for Animal Health, Greifswald-Insel Riems, Germany

**Keywords:** Vaccines, SARS-CoV-2

## Abstract

The ongoing SARS-CoV-2 pandemic necessitates the fast development of vaccines. Recently, viral mutants termed variants of concern (VOC) which may escape host immunity have emerged. The efficacy of spike encoding mRNA vaccines (CVnCoV and CV2CoV) against the ancestral strain and the VOC B.1.351 was tested in a K18-hACE2 transgenic mouse model. Naive mice and mice immunized with a formalin-inactivated SARS-CoV-2 preparation were used as controls. mRNA-immunized mice develop elevated SARS-CoV-2 RBD-specific antibody and neutralization titers which are readily detectable, but significantly reduced against VOC B.1.351. The mRNA vaccines fully protect from disease and mortality caused by either viral strain. SARS-CoV-2 remains undetected in swabs, lung, or brain in these groups. Despite lower neutralizing antibody titers compared to the ancestral strain BavPat1, CVnCoV and CV2CoV show complete disease protection against the novel VOC B.1.351 in our studies.

## Abstract (web summary)

Emerging SARS-CoV-2 variants with mutations in the spike protein raise concerns regarding vaccine efficacy. Here, the authors show that two spike encoding mRNA vaccines in preclinical and clinical development protect human ACE2 mice from the B.1.351 variant of concern and ancestral B BavPat1.

## Introduction

Coronavirus disease 2019 (COVID-19) severely affects human health and societies worldwide. It has accounted for >148 million morbidities and 3.1 million fatalities by end of April 2021 (World Health Organization (WHO), https://covid19.who.int). The responsible pathogen, severe acute respiratory syndrome coronavirus type 2 (SARS-CoV-2), has rapidly spread globally despite stringent intervention strategies^1^. To control pandemic spread and disease, vaccination is considered the most important and effective control measure^2^. Several vaccines based on mRNA technology or viral vectors are now authorized for emergency use and further products are in final licensing phases^3^. SARS-CoV-2 underwent adaptive mutations early during the pandemic, with the D614G variant becoming globally dominant at the beginning of 2020^4–6^. Viral evolution is a highly dynamic process that results in emergence of multiple, geographically distinct new variants, first identified in the UK (B1.1.7), South Africa (B.1.351), and Brazil (B.1.1.28; P1) (https://www.ecdc.europa.eu/en/publications-data/covid-19-risk-assessment-variants-vaccine-fourteenth-update-february-2021). These variants of concern (VOCs) acquired numerous mutations, particularly in the spike protein encoding gene (S), most frequently within the S1 and the receptor-binding domain (RBD)^7–9^. These mutations confer higher binding affinities and allow some VOCs to evade pre-existing immunity^10^, resulting in increased transmissibility, including epidemiologic scenarios where herd immunity was expected^11^. Whereas variant B.1.1.7 might still be efficiently neutralized by vaccination-elicited antibodies despite the RBD mutations^12–14^, variant B.1.351 showed a remarkable resistance to sera from vaccinated as well as from convalescent individuals^15–18^. VOCs that evade from efficient cross-neutralization may evolve into dominant strains and necessitate vaccine efficacy re-assessment. While indications for cross-neutralization exist, e.g., by sera from individuals vaccinated with SARS-CoV-2 mRNA vaccines^13,19^, in vivo data from experimental immunization/challenge studies in standardized animal models are pending. In this work, we investigated the efficacy of mRNA vaccines CVnCoV and CV2CoV against SARS-CoV-2 using an early B lineage 614G strain and the novel VOC B1.351 in a human ACE2 (hACE2) transgenic mouse model of severe COVID-19^20^. The choice for the variant B.1.351 was related to the observed immune-escape features with a reduced neutralization efficacy^10^ and decreased protective efficacy reported for a licensed vaccine^21^.

## Results

**Fig. 1:**
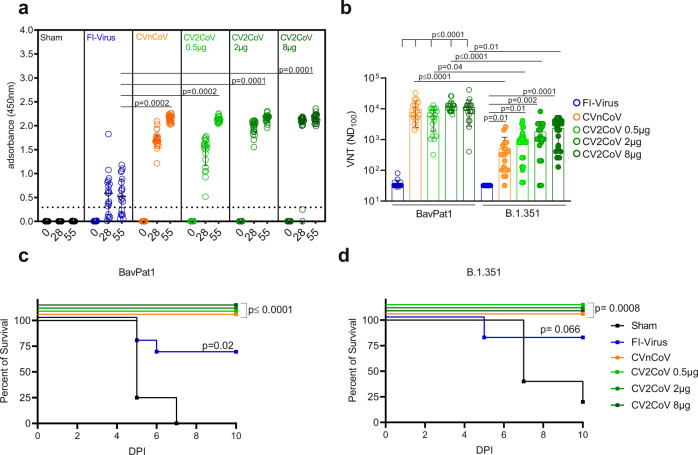
CVnCoV and CV2CoV protect K18-hACE2 mice against SARS-CoV-2 variants BavPat1 and B1.351. K18-hACE2 mice that were vaccinated with 8 µg CVnCoV (orange) or different concentrations of CV2CoV (0.5 µg (light green), 2 µg (green), or 8 µg (dark green)), received 10^6^ FI-Virus (blue) or NaCl (black) (sham) on days 0 and 28 followed by i.n. challenge with 10^5.9^ TCID_50_ of SARS-CoV-2 variant BavPat1 or 10^5.5^ TCID_50_ B1.351. **a** RBD ELISA with sera from K18-hACE2 mice on days 0, 28, and 55 of the respective groups: median and interquartile range are presented. Dashed line indicates threshold for positive anti-RBD antibody level. **b** Virus neutralization assay using day 55 sera from all the groups. Bars indicate mean with SD. **c**, **d** Survival curves (Kaplan–Meier) for K18-hACE2 mice from all the groups challenged either with BavPat1 (**c**) or B.1.351 (**d**) and followed up for 10 days post infection (DPI). **a**, **b** Each dot represents one individual mouse sample. Each sample was tested once (RBD ELISA) or in triplicates (VNT), and assays were repeated at least once. **c**, **d** Each line represents groups of mice as shown in **a**, **b** from a single experiment (*n* = 5 sham, *n* = 10 all other groups). *p* Values were determined by nonparametric one-way ANOVA and Dunn’s multiple comparisons test (**a**, **b**) or two-sided log-rank (Mantel–Cox) test (**c**, **d**). Differences were considered significant at *p* < 0.05 with exact *p* values displayed in the figure. Source data are provided as a Source data file.

We used the K18-hACE2 transgenic mouse model^22^ to determine the protective efficacy of two spike protein encoding mRNA vaccines, i.e., CVnCoV and CV2CoV, a clinical and preclinical stage vaccine, respectively. Both vaccines encode for the same protein, but differ in the non-coding regions of the mRNA. We chose a dose of 8 µg for CVnCoV, which conferred protection in hamsters^23^ and non-human primates (NHPs)^24^ in previous preclinical studies, and performed a dose titration ranging from 0.5 to 8 µg for the preclinical stage vaccine CV2CoV. Vaccine efficacy was tested against the ancestral SARS-CoV-2 B-lineage strain BavPat1 that closely matches the mRNA-encoded S protein and the heterologous VOC B.1.351 NW-RKI-I-0028. For immunization, CVnCoV vaccine, CV2CoV vaccine, or 20 µl of a formalin-inactivated and adjuvanted SARS-CoV-2-preparation (FI-Virus) were administered on days 0 and 28. Mice were challenged 4 weeks after boost vaccination with >10^5^ tissue culture infectious dose 50 (TCID_50_) of SARS-CoV-2 BavPat1 or B.1.351 NW-RKI-I-0028. A sham (NaCl) group served as non-vaccinated control (Fig. S1 and Table S1). Sera from all mRNA-vaccinated mice collected on days 28 and 55 showed a strong induction of anti-RBD total immunoglobulin (Ig), irrespective of the mRNA amount. Anti-RBD total Ig levels were significantly higher in sera from all mRNA-vaccinated groups compared to levels induced by the FI-Virus preparation (Fig. 1a). The strong induction of anti-RBD antibodies in the mRNA vaccine groups was reflected by high virus neutralization titers (VNTs). Of note, even a low dose of 0.5 µg of CV2CoV elicited high levels of humoral responses in K18-hACE2 transgenic mice. Sera from day 55 after immunization with CVnCoV or CV2CoV showed a significantly higher neutralizing capacity compared to sera from animals that had received the FI-Virus preparation. Importantly, neutralization of VOC B.1.351 was less effective compared to BavPat1 for the CVnCoV and CV2CoV groups, but far exceeded the values recorded for the FI-Virus group (Fig. 1b). Similar results were obtained with a surrogate VNT assay with post prime immunization sera collected at day 28 (Table S2). Overall, the tested mRNA vaccines induced robust antibody responses in a prime–boost regime, capable of efficiently neutralizing both BavPat1 and VOC B.1.351 NW-RKI-I-0028 in vitro.

Subsequently, the potential of CVnCoV and CV2CoV to protect from SARS-CoV-2 challenge infection was analyzed. Stocks of both challenge viruses were characterized by deep-sequencing demonstrating the characteristic mutations of VOC B.1.351, but no other relevant alterations (Fig. S3 and Table S3). Immunized K18-hACE2 mice were studied using a high-dose challenge model, which induces severe clinical disease resembling COVID-19 in humans^25^. In addition, mice develop severe encephalitis specific to this animal model^20^. On day 4, animals in the sham group started succumbing to the BavPat1 infection (Fig. 1c). B.1.351 infection led to a delayed onset of severe disease compared to BavPat1, with 20% survival on day 10 after inoculation (Fig. 1d). Thus, K18-hACE2 mice were highly susceptible to both SARS-CoV-2 variants. Importantly, vaccination with 8 µg CVnCoV or 0.5–8 µg CV2CoV resulted in complete protection (100% survival) against BavPat1 and B.1.351, with no significant weight loss or disease symptoms throughout the course of the challenge infection (Fig. 1c, d and Fig. S4). In contrast, prior administration of the FI-Virus preparation provided sub-optimal protection against either BavPat1 or B.1.351, resulting in weight loss and signs of distress (Fig. 1 and Fig. S3). Some of the FI-Virus-immunized animals experienced very early weight loss and disease signs after VOC B.1.351 challenge infection, earlier than sham groups. In conclusion, survival rates, body weight changes, and disease scores revealed complete protection by the CVnCoV and CV2CoV vaccines in K18-hACE2 mice against lethal SARS-CoV-2 challenge, including against VOC B.1.351.

**Fig. 2:**
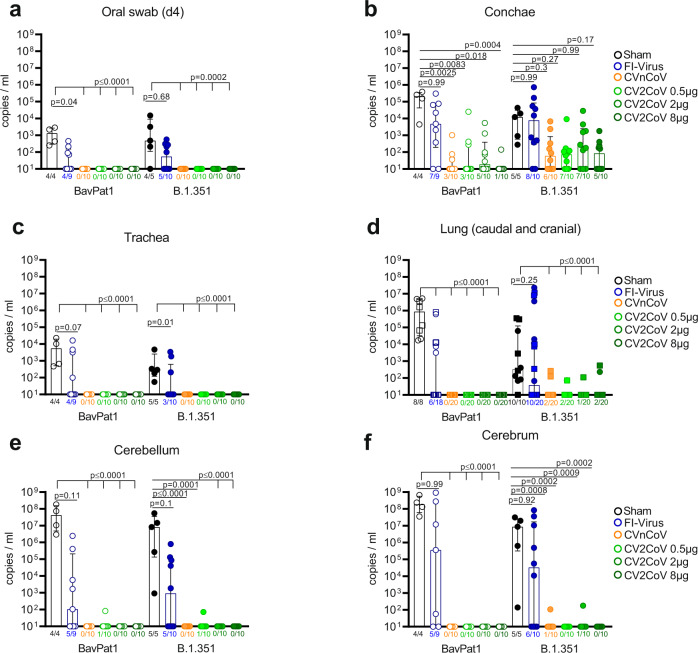
CVnCoV and CV2CoV prevent replication of SARS-CoV-2 variants BavPat1 and B.1.351 in K18-hACE2 mice. RT-qPCR for genomic RNA of SARS-CoV-2 was performed with **a** oral swab samples at day 4 or from organ samples of **b** the upper respiratory tract, **c**, **d** the lower respiratory tract (caudal lung = circle; cranial lung = squares), and **e**, **f** the brain at day 10 or at the humane endpoint. Mice that were vaccinated with 8 µg CVnCoV (orange) or different concentrations of CV2CoV (0.5 µg (light green), 2 µg (green) or 8 µg (dark green)), received 10^6^ FI-Virus (blue) or NaCl (black) (sham) on days 0 and 28 followed by i.n. challenge with 10^5.9^ TCID_50_ of SARS-CoV-2 variant BavPat1 or 10^5.5^ TCID_50_ B1.351. Each dot represents one individual mouse. Each sample was tested once, and assays were repeated at least once. *p* Values were determined by nonparametric one-way ANOVA and Dunn’s multiple comparisons test. Scatter plots are labeled with median (height of the bar) and interquartile range. Differences were considered significant at *p* < 0.05, with exact *p* values displayed in the figure. Source data are provided as a Source data file.

To investigate whether CVnCoV or CV2CoV vaccination prevented productive infection or dissemination of replicating SARS-CoV-2, we took oral swabs at 4 days post infection (dpi) to monitor viral RNA load in saliva. In the sham group, 4/4 and 4/5 samples were positive for viral genome after infection with BavPat1 or VOC B.1.351, respectively (Fig. 2a). FI-Virus administration prior to challenge did not significantly reduce viral genome load in saliva, with 40–60% of animals showing positive reverse transcription quantitative polymerase chain reaction (RT-qPCR) results on 4 dpi (Fig. 2a). In contrast, after CVnCoV or CV2CoV vaccination, no viral genomes were detected in oral swabs of either challenge groups irrespective of the vaccine and vaccine dose. Furthermore, the amount of subgenomic RNA (sgRNA) was determined from swabs that scored positive for total viral RNA. Two different assays indicated sgRNA only in a few samples from sham-vaccinated and challenged individual mice (Table S4). To further explore the prevention of viral replication following challenge, we determined viral load in the upper respiratory tract (URT) (conchae) and the lower respiratory tract (LRT) (trachea, caudal lung, and cranial lung), as well as in the central nervous system (brain, cerebellum/cerebrum) (Fig. 2b–f) in animals reaching the humane endpoint or at the day of termination (10 dpi). Similar to the quantitative RNA load results obtained from the oral swabs, the URT provided a niche for replication in both the sham and FI-Virus groups (Fig. 2b). In the CVnCoV and CV2CoV-vaccinated groups challenged with BavPat1, we observed a significant reduction of detectable viral replication in all groups with a maximum of 5/10 animals showing low genome copy numbers in the conchae. No animal in the LRT and only one sample from the brain was positive at a low level for SARS-CoV-2 genomic RNA, indicating complete protection from infection by BavPat1 in all the groups (Fig. 2c–f). The organ samples scoring positive for viral RNA were further evaluated using two distinct assays detecting sgRNAs. sgRNA was generally detected in samples that exhibited substantial total RNA loads (Table S5). More specifically, from CVnCoV- and CV2CoV-vaccinated mice that showed low levels of total RNA only a few single animals scored positive for sgRNA (Table S5). For VOC B.1.351, 5-7/10 CVnCoV or CV2CoV-vaccinated animals exhibited residual viral replication in the conchae. Here, viral levels were reduced without reaching statistical significance (Fig. 2b). Detection of sgRNA was limited to single individuals (Table S6). In contrast, both CVnCoV and CV2CoV almost completely prevented replication of this VOC in the LRT and the brain, with low viral copy numbers close to the limit of detection in the lung of 7/80 (40 cranial and 40 caudal)  specimens and only 1/40 and 2/40 animals in the cerebellum and cerebrum, respectively (Fig. 2c–f). No sgRNA could be amplified from these organs (Table S6). FI-Virus administration provided partial protection in the LRT in animals challenged with BavPat1, but not with VOC B.1.351, and did not significantly protect against viral replication in the cerebellum or cerebrum regardless of the SARS-CoV-2 variant (Fig. 2). These findings were verified by sgRNA detection (Tables S5 and S6). Of note, some of the animals receiving the FI-Virus preparation showed viral loads at the level of the sham group in the LRT (Fig. 2). In summary, aside conferring complete protection against lethal challenge with distinct SARS-CoV-2 lineages, CVnCoV and CV2CoV, at dosages ranging from 0.5 to 8 µg, prevented dissemination of SARS-CoV-2 from the inoculation site into other organs and provided solid protection against an ancestral SARS-CoV-2 and a VOC B.1.351 strain.

## Discussion

The emergence of new strains with immune-escape potential, such as the VOC of the B.1.351 lineage that appeared first in South Africa, are of great concern, since all available COVID-19 vaccines are based on the ancestral SARS-CoV-2 strains. We therefore tested two mRNA vaccines in different concentrations against a standard ancestral SARS-CoV-2 B lineage strain (BavPat1) in comparison to a VOC B.1.351 isolate in a transgenic mouse model.

Our data demonstrate that 8 µg CVnCoV and 0.5–8 µg CV2CoV fully protect mice against disease caused by two different SARS-CoV-2 variants. CVnCoV and CV2CoV vaccination, but not immunization with FI-Virus, rescued all transgenic mice from lethal infection caused by BavPat1 and VOC B.1.351 isolate NW-RKI-I-0028. The sub-optimal FI-Virus preparation reduced viral replication in the LRT solely after challenge with BavPat1, but showed no significant effect on viral dissemination as well as the viral genome loads in the URT. In contrast, both CVnCoV and CV2CoV immunization resulted in abundant RBD-specific and neutralizing antibodies and conferred complete and robust protection, including protection from viral replication in the lungs and brain. Only very limited viral replication was observed in the URT of mRNA-vaccinated animals challenged with VOC B.1.351. The relevance to disease transmission of this minimal viral replication in the conchae remains to be established. The reduced neutralizing capacity of sera from CVnCoV- and CV2CoV-vaccinated transgenic mice against VOC B.1.351, and the insufficient prevention of replication in the conchae, might reflect the currently detected transmission rates of this VOC in human populations previously exposed to the ancestral strain. Nevertheless, our study provides the first evidence for the efficacy of a vaccine to prevent disease and viral dissemination from the site of infection against an emerging SARS-CoV-2 variant in a sensitive, well-established, and accepted in vivo model. Of note, this model might not fully recapitulate viral dissemination into respiratory compartments in humans. The very high neutralizing titers against BavPat1 elicited by the mRNA immunization as well as the fold reduction recorded for VOC B.1.351 may be unique to the mouse model employed in this study and require validation in other experimental models. In addition, the variable neutralizing capacity of individual sera against VOC B.1.351 is in line with serological responses detected in human vaccinees^26^.

The pathophysiology of SARS-CoV-2 VOC infections remains largely unknown and detailed animal model data are missing. In our study, we observed a delayed course of disease in K18-hACE2 mice infected with a VOC B.1.351 strain and hypothesize that mutation accumulation might result in a changed in vivo phenotype. Short-term infections performed in hamsters have failed to detect diverging phenotypes in ancestral versus VOC lineages^27^, but comparable data sets about the complete course and replication in the URT are still pending for all VOCs in other animal models. These apparently discordant findings call for further pathological and immunological assessments of pathogenicity of emerging lineages.

Here we report full protection against a VOC by CVnCoV and CV2CoV immunization, associated with high anti-RBD and neutralizing antibodies. Whether antibodies alone were sufficient for the beneficial outcome remains to be further validated. Broad immune responses elicited by vaccines, including cellular responses in addition to neutralizing antibodies, antibody-dependent cytotoxicity, or antibody-mediated innate immune effector functions, could help explain the protection. Potent T cell immunity could ensure the success of an immunization when antibodies decline. CVnCoV vaccination was previously shown to induce Th1 immunity and trigger S-specific CD8^+^ T cell responses in mice^23^. Re-challenge studies in NHPs confirmed a role for CD8^+^ T cells in protection^28^. Although point mutations in the major histocompatibility complex-I-restricted viral epitopes could subvert CD8^+^ T cell surveillance^29^, the majority of SARS-CoV-2 T cell epitopes recognized by convalescent individuals or vaccinees immunized with licensed mRNA vaccines appear unaffected by unique VOC mutations^30^. These findings indicate a role of cellular immunity in defense against SARS-CoV-2. Here we observed solid protection against disease upon challenge infection with VOC B.1.351 after CVnCoV or CV2CoV vaccination, despite reduced virus-neutralizing titers. These observations suggest that either complementary immune mechanisms are effective or that residual virus-neutralizing titers against B.1.351 are sufficient for in vivo neutralization in this model. In line with this, it has recently been demonstrated in a NHP infection model that relatively low neutralizing antibody titers can protect from SARS-CoV-2-related clinical signs^28^. The precise contribution of various immune compartments to CVnCoV and CV2CoV efficacy requires further evaluation.

Our proof-of-principle study demonstrates that mRNA vaccines can protect hACE2 mice against disease caused by SARS-CoV-2 independent from the lineages or virus variants.

## Methods

### Ethics

The animal experiments were evaluated and approved by the ethics committee of the State Office of Agriculture, Food safety, and Fishery in Mecklenburg – Western Pomerania (LALLF M-V: 7221.3-1-055/20). All procedures using SARS-CoV-2 were carried out in approved biosafety level 3 facilities.

### Vaccination

Before challenge with SARS-CoV-2, mice were vaccinated prime day 0 and boost day 28 with either NaCl (sham), FI-Virus, or an mRNA vaccine (CVnCoV and CV2CoV) (Table S1).

The mRNA vaccines are based on the RNActive^®^ platform (claimed and described in, e.g., WO2002098443 and WO2012019780) and are comprised of a 5′ cap1 structure, a GC-enriched open reading frame (ORF), 3′ untranslated region (UTR), and a vector-encoded polyA stretch and do not include chemically modified nucleosides. CVnCoV contains parts of the 3′ UTR of the *Homo sapiens* alpha hemoglobin gene as 3′ UTR followed by a polyA stretch, a C30 stretch, and a histone stem loop. CV2CoV further comprises a 5′ UTR from the human hydroxysteroid 17-beta dehydrogenase 4 gene and a 3′ UTR from human proteasome 20S subunit beta 3 gene followed by a histone stem loop and a polyA stretch. Lipid nanoparticle (LNP) encapsulation of mRNA was performed by Acuitas Therapeutics (Vancouver, Canada). The LNPs used in this study are particles of ionizable amino lipid, phospholipid, cholesterol, and a PEGylated lipid. The mRNA-encoded protein is based on the spike glycoprotein of SARS-CoV-2 NCBI Reference Sequence NC_045512.2, GenBank accession number YP_009724390.1, and encodes for full-length S featuring K986P and V987P mutations.

For comparison to CVnCoV and CV2CoV, we used FI-Virus combined with Alhydrogel^®^ adjuvant. For this, 200 ml SARS-CoV-2 Germany/BavPat1/2020 (for details, see section “Challenge infection”) supernatant was concentrated using the PEG Virus Precipitation Kit (Biovision # BIV-K904) to a volume of 2 ml. Afterwards, this preparation was inactivated by formaldehyde (37%) at a dilution of 1:2000 at 37 °C for 24 h. Inactivation of the virus was confirmed by inoculation of VeroE6 cells. When no cytopathogenic effect (CPE) was detected, cell supernatants were passaged for three passages. For vaccination, freshly prepared stocks of 10^6^ TCID_50_ FI-Virus were mixed with 2% (final concentration) of Alhydrogel® in phosphate‐buffered saline (PBS). Prior vaccination, 20 µl of NaCl (sham control), FI-Virus, CVnCoV, or CV2CoV were loaded into single-use insulin syringe with an integrated needle (30 G) no longer than 2 h before injection. First, the mice were anesthetized by inhalation of isoflurane and the hind leg was shorn with an electric clipper. For all the groups, 20 µl of the preparation was administered intramuscularly into the M. tibialis (day 0 right leg or left day 28). Before animals were placed back into their cages, 100–140 µl blood samples were obtained by puncture of the V. facialis on days 0, 28, and 55. For blood collection, the animals remained anesthetized under isoflurane anesthesia (5 vol.%). All groups were monitored for side effects of the injection and were scored at 24 h post injection. The injection sides were slightly swollen in all the groups 24 h post vaccination, which resolved after 48–72 h.

### Serum collection

All blood samples were collected into Z-clot activator 200 µl microtube (Sarstedt). The samples were incubated at room temperature (RT) for 0.5–1 h and afterwards centrifuged for 5 min, 10,000 rcf, at RT. All serum samples were stored at <−70 °C.

### Virus preparation

SARS-CoV-2 Germany/BavPat1/2020 (BavPat1) (GISAID accession EPI_ISL_406862) was kindly provided by Bundeswehr Institute of Microbiology, Munich, Germany. SARS-CoV-2 hCoV-19/Germany/NW-RKI-I-0029/2020 B.1.351-linage or VOC 202012/02 (B.1.351) (GISAID accession EPI_ISL_803957) was kindly provided by Robert-Koch-Institut, Berlin, Germany. Virus stocks were propagated (three passages and two passages, respectively) on Vero E6 cells (Collection of Cell Lines in Veterinary Medicine CCLV-RIE 0929) using a mixture of equal volumes of Eagle MEM (Hanks’ balanced salts solution) and Eagle MEM (Earle’s balanced salts solution) supplemented with 2 mM L-Glutamine, nonessential amino acids adjusted to 850 mg/l, NaHCO_3_, 120 mg/l sodium pyruvate, and 10% fetal bovine serum, pH 7.2. The virus was harvested after 72 h, titrated on Vero E6 cells, and stored at −80 °C until further use.

### Sequencing of the viral genome

Full genome sequencing of SARS-CoV-2 B1.351 hCoV-19/Germany/NW-RKI-I-0029/2020 P2+1 (passage of hCoV-19/Germany/NW-RKI-I-0029/2020) was performed using high-throughput sequencing (HTS). For this purpose, RNA was extracted from cell culture supernatant using a combined TRIzol LS Reagent (Invitrogen, Waltham, MA, USA) and QIAamp RNeasy Mini Kit (Qiagen, Hilden, Germany) protocol. The resulting RNA extracts were subjected to library preparation as described in detail ^31^. The resulting library L4550 was quality-checked, quantified, and sequenced on the Ion Torrent S5XL platform on an Ion 530 sequencing chip using 400 bp chemistry.

The Genome Sequencer software suite (versions 2.6; Roche) was applied to execute reference mapping analyses. Since no complete whole-genome sequence was available of the original isolate (hCoV-19/Germany/NW-RKI-I-0029/2020|EPI_ISL_803957|2020-12-28), the genome sequence of SARS-CoV-2 B.1.351 isolate hCoV-19/Germany/BW-ChVir22275/2021|EPI_ISL_875344|2021-01-15 was used as initial reference. Subsequently, the mapping analysis was repeated with the obtained SARS-CoV-2 genome sequence as reference and the corresponding data set to compile the final SARS-CoV-2 genome sequence of the sample. This determined whole-genome sequence of sample L4550 was set as reference for variant calling. The Torrent Suite plugin Torrent variantCaller (version 5.12) was used to detect single-nucleotide polymorphism (SNP) variants (parameter settings: generic, S5/S5XL(530/540), somatic, low stringency, changed alignment arguments for the TMAP module from map 4 [default] to map1 map2). Identified SNP variants were visualized with Geneious Prime (10.2.3; Biomatters, Auckland, New Zealand) and compared with the SNP variants detected using the variant analysis tool implemented in Geneious Prime Molecular Biology and Sequence Analysis Software (version 10.2.3; (default settings, minimum variant frequency 0.02).

The SARS-CoV-2 genome sequence generated in this study is available under the accession number MZ433432.

### Challenge infection

Mice in groups of up to five animals were kept in individually ventilated cages (IVCs) for the entire study (Table S1). The animals were infected under short-term isoflurane inhalation anesthesia with 25 µl of either 10^5.875^ TCID_50_ SARS-CoV-2 BavPat1 (calculated from back-titration of the original material) or 10^5.5^ TCID_50_ SARS-CoV-2 B1.351 (calculated from back-titration of the original material) per animal. To prevent spill-over between different pairs, the IVCs were strictly separated in individual cage systems. During the entire study, all animals were offered water ad libitum and were fed and checked for clinical scores and body weight daily by animal caretakers and study researchers. Animal housing was performed with 20–24 °C temperature, 45–65% humidity; and 12-h dark/light cycle with 30 min of dawn. A oral swab sample of each animal was taken at 4 dpi under short-term isoflurane inhalation anesthesia. Animals with signs of severe clinical symptoms and/or body weight loss over 20% were euthanized before the end of the study. All animals were euthanized at day 10 post infection.

### RNA extraction and RT-qPCR

RNA from combined nasal/oral swabs and organ samples was extracted using the NucleoMag® VET Kit (Macherey-Nagel, Düren, Germany) in combination with a Biosprint 96 platform (Qiagen, Hilden, Germany). Each extracted sample was eluted in 100 µl. Viral RNA genome was detected and quantified by real-time RT-qPCR on a BioRad real-time CFX96 detection system (BioRad, Hercules, USA). Target sequence for amplification was the viral RNA-dependent RNA polymerase (WHO, https://www.who.int/docs/default-source/coronaviruse/real-time-rt-pcr-assays-for-the-detection-of-sars-cov-2-institut-pasteur-paris.pdf?sfvrsn=3662fcb6_2). Genome copies per µl RNA template were calculated based on a quantified standard RNA, where absolute quantification was done by the QX200 Droplet Digital PCR System in combination with the 1-Step RT-ddPCR Advanced Kit for Probes (BioRad, Hercules, USA). The limit of detection was calculated to be 10 copies per reaction.

### sgRNA RT-qPCR assays

Samples (swabs/organs) that tested positive for viral genomic RNA were evaluated using assays specifically detecting sgRNA. The assay detecting sgRNA of the E-gene established by Speranza et al.^32^ with the modification of omitting the ZEN™ quencher was used. In addition, a further assay was applied to detect sgRNA of the ORF7a, as Alexandersen and colleagues^33^ demonstrated that this sgRNA is more abundantly quantifiable. Primer and probe sequences of this assay are summarized in Table S7. The RT-qPCR reaction of both assays was prepared using the qScript XLT One-Step RT-qPCR ToughMix (QuantaBio, Beverly, MA, USA) in a volume of 12.5 µl including 1 µl of the respective FAM mix and 2.5 µl of extracted RNA. The reaction was performed for 10 min at 50 °C for reverse transcription, 1 min at 95 °C for activation, and 42 cycles of 10 s at 95 °C for denaturation, 10 s at 60 °C for annealing, and 20 s at 68 °C for elongation. Fluorescence was measured during the annealing phase. All RT-qPCRs were performed on a BioRad real-time CFX96 detection system.

### RBD antibody enzyme-linked immunosorbent assay (ELISA)

Sera were analyzed using an indirect multi-species ELISA based on the RBD of SARS-CoV-2^34^. For this, ELISA plates (Greiner Bio‐One GmbH) were coated with 100 ng/well the RBD overnight at 4 °C in 0.1 M carbonate buffer (1.59 g Na_2_CO_3_ and 2.93 g NaHCO_3_, ad. 1 L aqua dest., pH 9.6) or were treated with the coating buffer only. Afterwards, the plates were blocked for 1 h at 37 °C using 5% skim milk in PBS. Sera were pre‐diluted 1/100 in TBS-Tween (TBST) and incubated on the coated and uncoated wells for 1 h at RT. A multi‐species conjugate (SBVMILK; obtained from ID Screen® Schmallenberg virus Milk Indirect ELISA; IDvet) was diluted 1/80 and then added for 1 h at RT. Following the addition of tetramethylbenzidine substrate (IDEXX), the ELISA readings were taken at a wavelength of 450 nm on a Tecan Spectra Mini instrument (Tecan Group Ltd.). Between each step, the plates were washed three times with TBST. The absorbance was calculated by subtracting the optical density measured on the uncoated wells from the values obtained from the protein‐coated wells for the respective sample. Of note, the ELISA determines relative abundance of anti-RBD Ig levels and therefore does not allow a direct comparison between different studies.

### Virus neutralization test (VNT)

To evaluate specifically the presence of virus-neutralizing antibodies in serum samples (pre-challenge), we performed a VNT. Therefore, sera were pre-diluted 1/16 or 1/32 with Dulbecco’s modified Eagle’s medium (DMEM) in a 96-well deep well master plate. Three times 100 µl, representing three technical replicates, of this pre-dilution were transferred into a 96-well plate. A log2 dilution was conducted by passaging 50 µl of the serum dilution in 50 µl DMEM, leaving 50 µl of sera dilution in each well. Subsequently, 50 µl of the respective SARS-CoV-2 (BavPat1 or B.1.351) virus dilution (100 TCID_50_/well) was added to each well and incubated for 1 h at 37 °C. Lastly, 100 µl of trypsinated VeroE6 cells (cells of one confluent TC175 flask per 100 ml) in DMEM with 1% penicillin/streptomycin supplementation was added to each well. After 72 h incubation at 37 °C, the cells were evaluated by light microscopy for a specific CPE. A serum dilution was counted as neutralizing in the case no specific CPE was visible. The virus titer was confirmed by virus titration; positive and negative serum samples were included.

### SARS-CoV-2 surrogate VNT

Individual serum samples obtained after prime, but before boost vaccination, were evaluated by the use of a virus neutralization surrogate assay (GenScript Biotech, Leiden, The Netherlands). Number of samples matches the number of samples presented in Fig. 1. The assay was used according to the manufacturer’s instructions.

### Reporting summary

Further information on research design is available in the Nature Research Reporting Summary linked to this article.

## Supplementary information

Supplementary information

Reporting Summary

## Data Availability

The authors declare that the data supporting the findings of this study are available within the paper and its supplementary information files and are available from the corresponding authors upon reasonable request. The SARS-CoV-2 genome sequence generated in this study is available under the accession number MZ433432. The following sequence data was used: SARS-CoV-2 NCBI Reference Sequence NC_045512.2, GenBank accession number YP_009724390.1; SARS-CoV-2 Germany/BavPat1/2020 (BavPat1) (GISAID accession EPI_ISL_406862); SARS-CoV-2 hCoV-19/Germany/NW-RKI-I-0029/2020 (GISAID accession EPI_ISL_803957). All non-commercial materials generated during the current study are available from the corresponding authors under an MTA with Friedrich-Loeffler-Institut. Source data are provided with this paper.

## Source data

Source Data
