# Supplementary material for: CVnCoV and CV2CoV protect human ACE2 transgenic mice from ancestral B BavPat1 and emerging B.1.351 SARS-CoV-2
Source: Nat Commun. 2021 Jun 30;12:4048. doi: 10.1038/s41467-021-24339-7 (PMC8245475; doi:10.1038/s41467-021-24339-7)
Supplement: Supplementary file 1 — Supplementary information [file 41467_2021_24339_MOESM1_ESM.pdf]

Supplemental Material

CVnCoV and CV2CoV protect human ACE2 transgenic mice from ancestral B BavPat1 and emerging B.1.351 SARS-CoV-2

Table S1: Experimental and control groups.

group	No. of animals	Vaccine	Dose, volume, route	Dosing	Serum collection	Challenge infection		End of the study
I	K18-hACE2 mice Female n=10	CVnCoV	8 µg, 20 µl, i.m.	d0, 28	d0, d28, d55	BavPat1		d69 (10dpc)
II			8 µg, 20 µl, i.m.				B1.351	
III		CV2CoV	0.5 µg, 20 µl, i.m.			BavPat1		
IV			0.5 µg, 20 µl, i.m.				B1.351	
V		CV2CoV	2 µg, 20 µl, i.m.			BavPat1		
VI			2 µg, 20 µl, i.m.				B1.351	
VII		CV2CoV	8 µg, 20 µl, i.m.			BavPat1		
VIII			8 µg, 20 µl, i.m.				B1.351	
IX		Formalin-inactivated virus +Alum	10^{6} $TCID_{50}$, 20µl, i.m.			BavPat1		
X			10^{6} $TCID_{50}$, 20µl, i.m				B1.351	
XI	n=5	Sham (NaCl)	-, 20 µl, i.m.			BavPat1		
XII			-, 20 µl, i.m.				B1.351	

Table S2. sVNT results of serum samples from day 28

**Group**	**Median**	**IQR**	**p-value**
Sham	6.83	3.69-7.94	N/A
FI -Virus	55.68	26.01-71.69	N/A
CVnCoV	94.83	93.89-95.27	0.0017
CV2CoV 0.5µg	93	88.69-95.34	0.03
CV2CoV 2µg	96.01	95.52-96.27	<0.0001
CV2CoV 8µg	96.46	96.09-96.68	<0.0001

IQR= interquartile range; P-values compare all RNA vaccine groups to the FI-Virus group and were determined by nonparametric one-way ANOVA and Dunn’s multiple comparisons test

Source data are provided as a Source Data file.

Table S3. Sequencing coverage of L4550 for mutations typically found in the spike protein gene of B.1.351 variants and the spike protein polybasic cleavage site.

B.1.351 mutations Spike	Coverage	Variant
**L18F**	8,584 reads	None
**D80A**	6,361 reads	None
**D215G**	9,898 reads	None
**Deletion 241-243**	(9628 reads)	None
**K417N**	8,606 reads	None
**E484K**	9,094 reads	None
**N501Y**	9,868 reads	None
**A701V**	14,038 reads	None
**Cleavage site**	17,730 reads	None

Table S4: Viral RNA genome content detected in swab samples (4 dpi), expressed as Cq values for total RNA and subgenomic RNA. This table includes samples that were positive for total RNA, all further swab samples scored negative.

**Vaccine**	**Challenge**	**ID**	**Genomic RNA**	**Subgenomic RNA**	
				**sgRNA E**	**sgRNA ORF7a**
FI virus	BavPat1	FU5923	36.6	nd	nd
		QE7311	35.4	nd	nd
		VI9303	38.3	nd	nd
		KZ7407	37.9	nd	nd
	B.1.351.	QE7312	37.8	nd	nd
		QE7300	36.0	nd	nd
		VK1621	36.2	nd	nd
		KZ7411	36.6	nd	nd
		VI9302	35.0	nd	nd
sham	BavPat1	AO8979	32.9	nd	nd
		AO8971	35.4	nd	nd
		AO8960	32.5	nd	nd
		VK1079	36.3	nd	nd
	B.1.351.	KZ7412	35.3	nd	nd
		VI9315	32.3	nd	36.7
		VI9305	29.9	37.1	36.2
		KZ7416	36.6	nd	nd

nd: not detected

Table S5: Viral RNA genome content detected in organ samples (10 dpi or indicated dpi) from mice challenged with the isolate BavPat1 (homologous spike), expressed as Cq value for total RNA and subgenomic RNA. The table includes samples that were positive for total genomic RNA (Cq <40), while all further organ samples scored negative.

**Vaccine**	**Challenge**	**ID**	**Organ**	**Genomic RNA**	**Subgenomic RNA**	
					**sgRNA E**	**sgRNA ORF7a**
FI virus	BavPat1	FU5923	Cerebellum	39.6	nd	nd
			Cerebrum	25.7	29.3	26.2
			Nasal conchae	26.4	32.7	31.4
			Nasal conchae	33.7	nd	nd
		QE7309	Cerebellum	34.1	nd	33.2
			Cerebrum	25.6	30.7	26.7
			Nasal conchae	36.5	nd	nd
		KZ7402	Nasal conchae	28.1	35.8	33.2
		VI9304	Cerebrum	39.5	nd	nd
			Trachea	32.5	39.1	38.4
		VI9303 (6dpc)	Cerebellum	31.4	38.7	32.9
			Cerebrum	19.4	24.7	21.4
			Nasal conchae	27.6	37.1	32.5
			Trachea	37.8	nd	nd
			Lung caudal	32.3	38.5	35.7
			Lung cranial	30.1	36.9	34.2
		QE7311 (5dpc)	Cerebellum	22.6	27.5	25.3
			Cerebrum	15	20.7	18.2
			Nasal conchae	32.6	nd	36.7
			Trachea	30.7	37.6	38.8
			Lung caudal	31.9	nd	37.3
			Lung cranial	31.1	nd	34.1
		VK1634 (5dpc)	Cerebellum	28.5	30.8	28.1
			Cerebrum	13.1	18.1	15.5
			Nasal conchae	30.2	nd	37.7
			Trachea	33.3	nd	36
			Lung caudal	24.8	30.3	27.4
			Lung cranial	24.4	29.8	27
sham	BavPat1	AO8971 (7dpc)	Cerebellum	20.3	24.3	21.4
			Cerebrum	18.3	22.7	19.5
			Nasal conchae	32.8	nd	nd
			Trachea	36.4	nd	nd
			Lung caudal	30.3	nd	nd
			Lung cranial	27.3	32.1	29.4
		VK1079 (5dpc)	Cerebellum	22.3	35.4	23.2
			Cerebrum	13.6	18.6	16.4
			Nasal conchae	26.2	34.7	30.4
			Trachea	31.4	36.9	34.7
			Lung caudal	21.9	27.1	23.5
			Lung cranial	30.6	nd	33.8
		AO8960 (5dpc)	Cerebellum	17.2	20.4	18.5
			Cerebrum	16.2	18.1	5.9
			Nasal conchae	27	30.3	29
			Trachea	35.8	nd	40.5
			Lung caudal	21.4	26.1	23.1
			Lung cranial	23.3	28	25.4
		AO8979 (5dpc)	Cerebellum	15.7	19.9	17
			Cerebrum	15.3	19.3	16.9
			Nasal Conchae	25.6	31.5	29.3
			Trachea	30.1	38.1	34.4

			Lung caudal	21.5	25.3	22.8
			Lung cranial	28.4	33.1	30
CVnCoV	BavPat1	QE7304	Nasal conchae	38.2	nd	nd
		FT9301	Nasal conchae	35.3	nd	nd
CV2CoV 8µg	BavPat1	KY5549	Nasal conchae	38.7	nd	nd
CV2CoV 2µg	BavPat1	VI9314	Nasal conchae	39.6	nd	nd
		VI9316	Trachea	37.7	nd	nd
		KZ7417	Nasal conchae	32.6	39.6	nd
		VK1078	Nasal conchae	35	nd	nd
		VK1072	Nasal conchae	39.1	nd	nd
CV2CoV 0.5µg	BavPat1	FT9319	Nasal conchae	36.1	nd	nd
		FT9307	Cerebellum	38.9	nd	nd
			Nasal conchae	36.3	nd	nd
		KY5552	Nasal conchae	30.5	37.1	36.2

nd: not detected

Table S6: Viral RNA genome content detected in organ samples (10 dpi or indicated) from mice challenged with the B.1.351. isolate (heterologous spike), expressed as Cq value for total genomic RNA and subgenomic RNA. This table includes samples that resulted positive for total RNA (Cq <40), while all further organ samples scored negative.

Vaccine	Challenge	ID	Organ	Genomic RNA	Subgenomic RNA	
					sgRNA E	sgRNA ORF7a
FI virus	B.1.351.	QE7302	Cerebrum	29	35.8	31.1
			Nasal conchae	29.7	39.2	34.6
			Lung cranial	37.4	nd	nd
		VK1631	Nasal conchae	36.8	nd	nd
		QE7312	Cerebellum	29.6	39.7	31.9
			Cerebrum	31.7	nd	33.8
			Trachea	38.2	nd	nd
			Lung caudal	30.7	39.1	35.2
			Lung cranial	31.8	39.7	35.5
		KZ7403	Nasal conchae	29.1	nd	nd
		VI9309	Cerebellum	33.9	nd	34.8
			Cerebrum	25.1	31.2	28.3
			Nasal conchae	36.5	nd	nd
			Lung caudal	39.2	nd	nd
		KZ7413	Cerebellum	27.9	nd	31.4
			Cerebrum	18.3	26.3	23.8
			Nasal conchae	35.4	nd	nd
		KZ7411	Nasal conchae	30.7	38.2	nd
			Lung caudal	31.1	36.6	35.5
			Lung cranial	31.7	38.2	35.3
		QE7300 (5dpc)	Cerebellum	27.3	nd	30.4
			Cerebrum	20.3	25.7	22.4
			Nasal conchae	26.9	32.8	31.9
			Trachea	34.1	nd	37.6
			Lung caudal	22	25.5	24.1
			Lung cranial	20.7	25.8	24.5
		VI9302 (5dpc)	Cerebellum	24.4	29.1	26
			Cerebrum	16.9	22.7	20
			Nasal conchae	24.5	29.8	28.9
			Trachea	33.2	nd	nd
			Lung caudal	19	22.8	21.1
			Lung cranial	20.1	23.9	22.3
sham	B.1.351.	VI9305	Cerebellum	26.5	33.4	28.4
			Cerebrum	25.1	30	26.5
			Nasal conchae	31.5	36.4	36.6
			Trachea	38.3	nd	nd
			Lung caudal	39.6	-	-
		KZ7416	Cerebellum	35.8	nd	nd
			Cerebrum	38.9	nd	nd
			Nasal conchae	30.7	nd	34.8
			Trachea	37.7	nd	nd

			Lung caudal	28.9	33.9	31.6
			Lung cranial	26.3	32	29.4
		VI9306 (7dpc)	Cerebellum	19.7	23.9	20.1
			Cerebrum	19.3	24.2	20.6
			Nasal conchae	35.3	nd	nd
			Trachea	39.8	-	-
			Lung caudal	38.4	nd	nd
			Lung cranial	37.2	nd	39.8
		KZ7412 (7dpc)	Cerebellum	20.7	24.5	22.3
			Cerebrum	18.5	23.5	21.2
			Nasal conchae	29.1	36.3	34.1
			Trachea	32.7	nd	39.4
			Lung caudal	36.6	nd	nd
			Lung cranial	33.5	nd	38.4
		VI9315 (7dpc)	Cerebellum	17.6	21.2	18.2
			Cerebrum	20.6	25.9	23.1
			Nasal conchae	37.2	nd	nd
			Trachea	36.9	nd	nd
			Lung caudal	39.4	nd	nd
			Lung cranial	25.7	30	28.5
CVnCoV	B.1.351.	KY5553	Nasal conchae	39.1	nd	nd
		KY5554	Nasal conchae	37	nd	nd
		VK1636	Nasal conchae	34.5	nd	nd
		VK1633	Cerebrum	39.6	nd	nd
			Nasal conchae	32.7	nd	37.7
			Lung cranial	38.1	nd	nd
		QE7314	Nasal conchae	39	nd	nd
			Lung cranial	39.4	nd	nd
CV2CoV 8µg	B.1.351.	KZ6841	Nasal conchae	38	nd	nd
		FT9314	Nasal conchae	38.2	nd	nd
		KZ6850	Nasal conchae	34.3	nd	nd
		AO5179	Nasal conchae	37.5	nd	nd
		FT9309	Nasal conchae	36.7	nd	nd
			Lung caudal	37.6	nd	nd
			Lung cranial	36.2	nd	nd
CV2CoV 2µg	B.1.351.	AO8968	Nasal conchae	37.3	nd	nd
		VK1074	Nasal conchae	38	nd	nd
		VK1076	Nasal conchae	32.9	nd	nd
		VK1067	Nasal conchae	38.5	nd	nd
		AO8965	Nasal conchae	34.6	nd	nd
			Lung cranial	38.8	nd	nd

		AO8977	Nasal conchae	34.3	nd	nd
		VK1077	Cerebrum	38	nd	nd
			Nasal conchae	29.9	36.9	35.8
CV2CoV 0.5µg	B.1.351.	AO5160	Nasal conchae	38.5	nd	nd
		KY5557	Cerebellum	39.1	nd	nd
		QE6769	Nasal conchae	38.3	nd	nd
			Lung caudal	39.1	nd	nd
			Lung cranial	39.1	nd	nd
		AO5163	Nasal conchae	39.3	nd	nd
		FU7019	Nasal conchae	38.5	nd	nd
		AO5166	Nasal conchae	31.7	nd	38.6
		AO5177	Nasal conchae	39.2	nd	nd

nd: not detected; -: not tested

Table S7: Primer and probes used to detect genomic RNA and sgRNA from SARS-CoV-2 ORF7a

	**sequence (5´-3´)**	**position***	amplicon
RdRp gene / nCoV_IP4 (https://www.who.int/docs/default-source/coronaviruse/real-time-rt-pcr-assays-for-the-detection-of-sars-cov-2-institut-pasteur-paris.pdf?sfvrsn=3662fcb6_2)			
nCoV_IP4-14059Fw	GGT AAC TGG TAT GAT TTC G	14080-14098	107 bp
nCoV_IP4-14146Rv	CTG GTC AAG GTT AAT ATA GG	14186-14167	
nCoV_IP4-14084Probe(+)	FAM-TCATACAAACCACGCCAGG-BHQ-1	14105-14123	
sgRNA ORF7a			
sgRNA-Lead-2F	CCA GGT AAC AAA CCA ACC AAC T	20-41	133bp
sgRNA-ORF7a-2R	ACC TCT AAC ACA CTC TTG GTA G	27471-27450	
sgRNA-ORF7a-2FAM	FAM-TCT TGG CAC TGA TAA CAC TCG CTA CT-BHQ1	27410-27435	

*position according to accession number NC_045512

Fig. S1

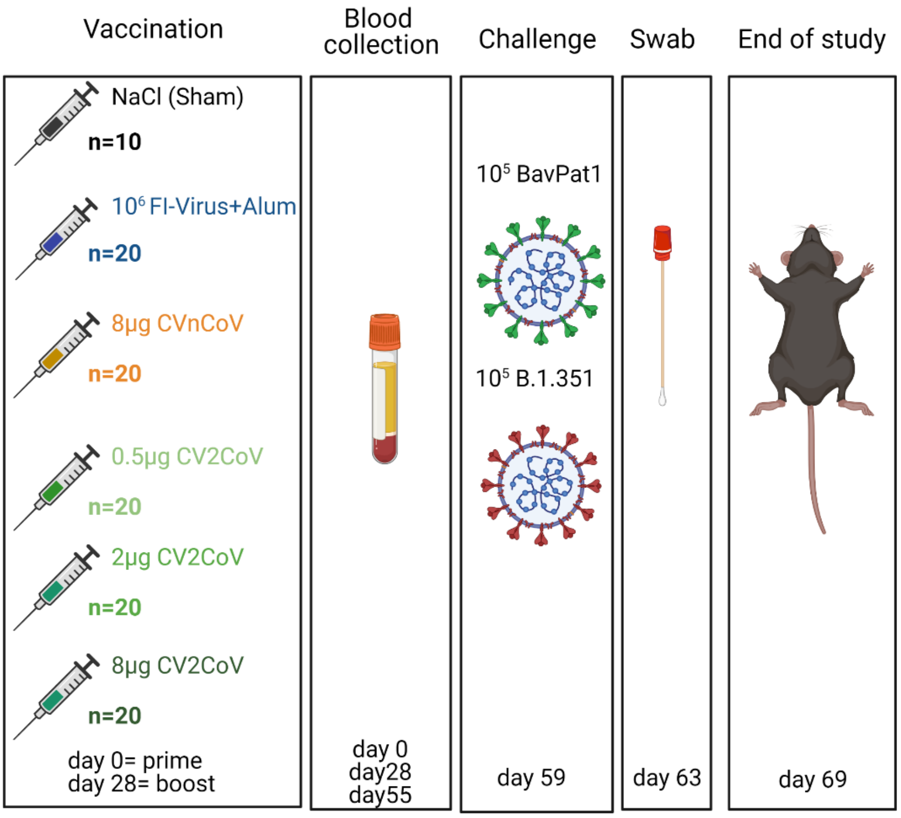

Fig.S1. Experimental design. K18-hACE2 mice were either vaccinated at day 0 (prime) and day 28 (boost) i.m. with 20µl of 8µg CVnCoV, 0.5µg CV2CoV, 2µg CV2CoV, 8µg CV2CoV or received 20µl NaCl (Sham) or 20µl of 10^6 $TCID_{50}$ FI-Virus+2%Alhydrogel® in PBS which served as control groups. Blood samples were collected at day 0, day 28 and day 55. Mice were either challenged with 10^5 SARS-CoV-2 BavPat1 (Sham n=4, FI-Virus n=10, CVnCoV n=10) or 10^5 B.1.351 (Sham n=5, FI-Virus n=10, CVnCoV n=10) at day 59 for a total of 10 days. An oral swab was taken at day 4. Viral load was determined in selected organs at day 10 or when animals reached the humane endpoint of the study. Image was generated using the illustration software Biorender (Biorender.com)

Fig. S2

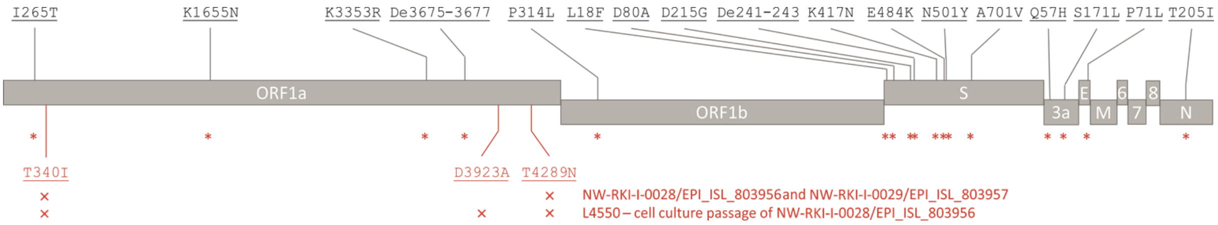

Fig.S2 Cell culture passage of the VOC B.1.351 NW-RKI-I-0028 strain did not alter genome sequence of the challenge virus stock L4550. All characteristic mutations of VOC B.1.351 (upper panel) were detected in the deeply sequenced stock of the challenge virus B.1.351 NW-RKI-I-0028 (L4550) as marked by red asterisks. Additional alterations for the B.1.351 NW-RKI-I-0028 and NW-RKI-I-0029 strains and L4550 cell culture passage are shown (lower panel). Mean coverage of the virus genome sequence of L4550 was 13,184 (± 7,958.4) reads. No single nucleotide polymorphism variants were detected in the mutations (L18F, D80A, D215G, deletion De241-243, K417N, E484K, N501Y, A701V) and in the cleavage site of the spike glycoprotein.

Fig. S3

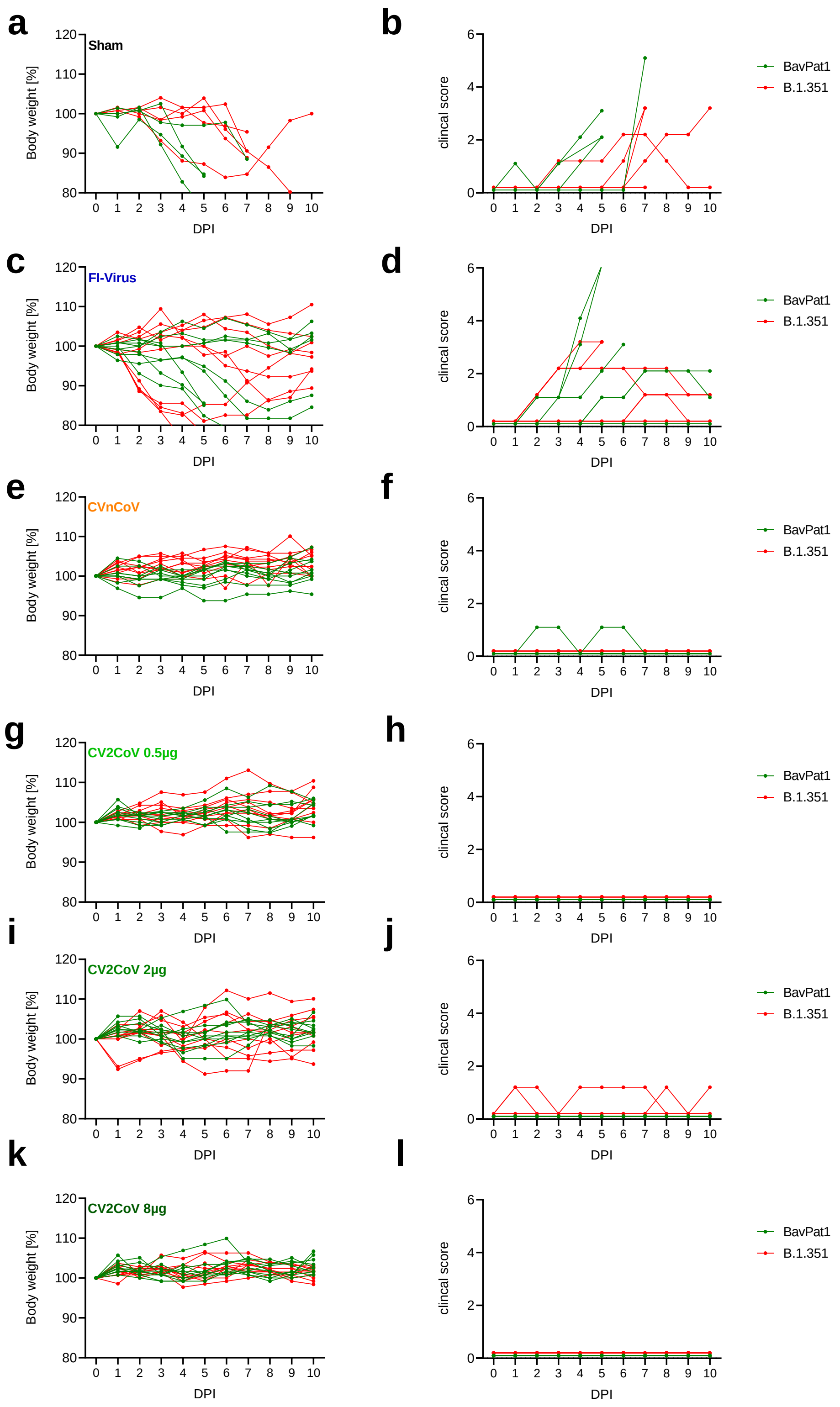

Fig. S3 CVnCoV and CV2CoV protect K18-hACE2 mice against SARS-CoV-2 variants BavPat1 and B.1.351. K18-hACE2 mice were vaccinated and challenged with SARS-CoV-2 variants BavPat1 or B.1.351 as indicated in Fig. 1. (**A, C, E, G, I, K**) Body weight and (**B, D, F, H, J, L**) clinical score

for K18-hACE2 mice in the (**A** and **B**) SHAM, (**C** and **D**) FI-Virus, (**E** and **F**) CVnCoV, (**G** and **H**) CV2CoV 0.5µg, (**G** and **H**) CV2CoV 2µg, and (**G** and **H**) CV2CoV 8µg groups was monitored daily. Lines represent individual animals over the course of the experiment (BavPat1 = green; B.1.351 = red).
