# Supplementary material for: CVnCoV and CV2CoV protect human ACE2 transgenic mice from ancestral B BavPat1 and emerging B.1.351 SARS-CoV-2
Source: Nat Commun. 2021 Jun 30;12:4048. doi: 10.1038/s41467-021-24339-7 (PMC8245475; doi:10.1038/s41467-021-24339-7)
Supplement: Supplementary file 2 — Reporting Summary [file 41467_2021_24339_MOESM2_ESM.pdf]

nature research

Corresponding author(s): Martin Beer, Anca Dorhoi

Last updated by author(s): Jun 1, 2021

Reporting Summary

Nature Research wishes to improve the reproducibility of the work that we publish. This form provides structure for consistency and transparency in reporting. For further information on Nature Research policies, see our Editorial Policies and the Editorial Policy Checklist.

Statistics

For all statistical analyses, confirm that the following items are present in the figure legend, table legend, main text, or Methods section.

n/a	Confirmed	
☐	☒	The exact sample size (*n*) for each experimental group/condition, given as a discrete number and unit of measurement
☐	☒	A statement on whether measurements were taken from distinct samples or whether the same sample was measured repeatedly
☐	☒	The statistical test(s) used AND whether they are one- or two-sided<br>*Only common tests should be described solely by name; describe more complex techniques in the Methods section.*
☒	☐	A description of all covariates tested
☒	☐	A description of any assumptions or corrections, such as tests of normality and adjustment for multiple comparisons
☐	☒	A full description of the statistical parameters including central tendency (e.g. means) or other basic estimates (e.g. regression coefficient) AND variation (e.g. standard deviation) or associated estimates of uncertainty (e.g. confidence intervals)
☐	☒	For null hypothesis testing, the test statistic (e.g. *F*, *t*, *r*) with confidence intervals, effect sizes, degrees of freedom and *P* value noted<br>*Give P values as exact values whenever suitable.*
☒	☐	For Bayesian analysis, information on the choice of priors and Markov chain Monte Carlo settings
☒	☐	For hierarchical and complex designs, identification of the appropriate level for tests and full reporting of outcomes
☒	☐	Estimates of effect sizes (e.g. Cohen's *d*, Pearson's *r*), indicating how they were calculated

Our web collection on statistics for biologists contains articles on many of the points above.

Software and code

Policy information about availability of computer code

Data collection	NCBI GenBank and GISAID e.V. EpiCov databases for SARS-CoV-2 sequence comparisons, no software was used for data collection
Data analysis	Geneious Prime Molecular Biology and Sequence Analysis Software (version 10.2.3);Genome Sequencer software suite (versions 2.6; Roche); GraphPad Prism 9; Microsoft excel from Microsoft Office Professional Plus 2019 Version 1808

For manuscripts utilizing custom algorithms or software that are central to the research but not yet described in published literature, software must be made available to editors and reviewers. We strongly encourage code deposition in a community repository (e.g. GitHub). See the Nature Research guidelines for submitting code & software for further information.

Data

Policy information about availability of data

All manuscripts must include a data availability statement. This statement should provide the following information, where applicable:

- Accession codes, unique identifiers, or web links for publicly available datasets
- A list of figures that have associated raw data
- A description of any restrictions on data availability

Sequence data generated in this study are availabe at the European Nucleotide Archive (PRJEB43810);
The authors declare that most data supporting the findings of this study are available within the paper and its supplementary information files. Further data that support the findings of this study are available from the corresponding authors upon reasonable request.
SARS-CoV-2 NCBI Reference Sequence NC_045512.2, GenBank accession number YP_009724390.1
SARS-CoV-2 Germany/BavPat1/2020 (BavPat1) (GISAID accession EPI_ISL_406862)
SARS-CoV-2 hCoV-19/Germany/NW-RKI-I-0029/2020 (GISAID accession EPI_ISL_803957)

Field-specific reporting

Please select the one below that is the best fit for your research. If you are not sure, read the appropriate sections before making your selection.

[x] Life sciences [ ] Behavioural & social sciences [ ] Ecological, evolutionary & environmental sciences

For a reference copy of the document with all sections, see nature.com/documents/nr-reporting-summary-flat.pdf

Life sciences study design

All studies must disclose on these points even when the disclosure is negative.

Sample size	Assessment of animals in each group was based on a one-sided Fisher exact test with a significance level of 5% and a power of 80% (Power 1-ß = 80%) to allow detection of a significant reduction of the viral load by 50% between Sham and immunized groups.
Data exclusions	No data were excluded.
Replication	Elisa-tests and RT-qPCR (three assays from the identical extracted NA) were replicated once. VNTs were assessed in triplicates. The animal experiment was performed once with the following animal numbers: vaccinated groups N=10, Sham controls N=5. Replication of experiments were successfull.
Randomization	K18-hACE2 mice were randomly assigned to the groups outlined in the manuscript (Sham, FI-Virus, CVnCoV, CV2CoV 0.5-8µg). All generated samples were tested individually and all results were included, therefore randomization of samples was not relevant.
Blinding	This pre-clinical research was performed without blinding in the animal facility as there was a sufficient time gap between application of the treatment and the outcome recording/assessment, but blinding was applied for sample analyses.

Reporting for specific materials, systems and methods

We require information from authors about some types of materials, experimental systems and methods used in many studies. Here, indicate whether each material, system or method listed is relevant to your study. If you are not sure if a list item applies to your research, read the appropriate section before selecting a response.

Materials & experimental systems

n/a	Involved in the study	
[x]	[ ]	Antibodies
[ ]	[x]	Eukaryotic cell lines
[x]	[ ]	Palaeontology and archaeology
[ ]	[x]	Animals and other organisms
[x]	[ ]	Human research participants
[x]	[ ]	Clinical data
[x]	[ ]	Dual use research of concern

Methods

n/a	Involved in the study	
[x]	[ ]	ChIP-seq
[x]	[ ]	Flow cytometry
[x]	[ ]	MRI-based neuroimaging

Eukaryotic cell lines

Policy information about cell lines

Cell line source(s)	Vero E6 (Collection of Cell Lines in Veterinary Medicine; CCLV FLI Insel Riems, Germany) from ATCC Vero C1008
Authentication	The cell line was not authenticated.
Mycoplasma contamination	All cell lines were quality screened and were tested free for mycoplasma.
Commonly misidentified lines (See ICLAC register)	No misidentified cell lines were used.

Animals and other organisms

Policy information about studies involving animals; ARRIVE guidelines recommended for reporting animal research

Laboratory animals	9 week old, female K18-hACE2 transgenic mice expressing human ACE2 (The Jackson Laboratory);<br>20-24°C temperature, 45-65 % humidity; 12h dark/light cycle with 30 minutes of dawn
Wild animals	No wild animals were used in the study.
Field-collected samples	No field-collected samples were used in the study.
Ethics oversight	Ethics approval by the ethics committee of the State Office of Agriculture, Food safety, and Fishery in Mecklenburg – Western Pomerania (LALLF M-V: LVL MV/TSD/7221.3-1-055/20).<br>All procedures using SARS-CoV-2 were carried out in approved biosafety level 3 (BSL3) facilities

Note that full information on the approval of the study protocol must also be provided in the manuscript.
